# Mining chemical information in Swedish wastewaters for simultaneous assessment of population consumption, treatment efficiency and environmental discharge of illicit drugs

**DOI:** 10.1038/s41598-021-92915-4

**Published:** 2021-06-29

**Authors:** Inga Haalck, Paul Löffler, Christine Baduel, Karin Wiberg, Lutz Ahrens, Foon Yin Lai

**Affiliations:** 1 Department of Aquatic Sciences and Assessment, Swedish University of Agricultural Sciences (SLU), Box 7050, 75007 Uppsala, Sweden; 2 University Grenoble Alpes, IRD, CNRS, Grenoble INP, IGE, Grenoble, France

**Keywords:** Environmental chemistry, Environmental impact, Population screening

## Abstract

Consumption of illicit drugs poses health risks to the public and environment. Knowledge on their usage helps better implementations of intervention strategies to reduce drug-related harms in the society and also policies to limit their releases as emerging contaminants to recipient environments. This study aimed to investigate from the daily consumption to treatment efficiency and subsequent discharge of illicit drugs by the Swedish urban populations based on simultaneous collection and analysis of influent and effluent wastewater. Two different weekly monitoring campaigns showed similar drug prevalence in Stockholm and Uppsala, with amphetamine as the most popular drug. Almost all target drug residues were still measurable in effluent wastewater. High removal efficiencies (> 94%) were observed for amphetamine, cocaine and benzoylecgonine, whereas ketamine, 3,4-methylenedioxymethamphetamine (MDMA), mephedrone and methamphetamine were the least removed substances (< 64%), with the highest discharge observed for MDMA in both catchments (~ 3.0 g/day in Uppsala; ~ 18 g/day in Stockholm). Our study provides new insights into short-term changes in the use and related discharge of illicit drugs by urban populations. Such wastewater monitoring can provide useful information to public health, forensic and environmental authorities in planning future intervention and regulation policies.

## Introduction

Consumption of illicit drugs poses a threat to public health in different countries, leading to chronic and acute impacts on population well-being, such as transmitting infectious diseases (e.g. drug injection with sharing tools), drug-related deaths (e.g. overdose), and criminal activities (e.g. violence, drug selling)^1,2^. Although drug consumption in Sweden has been found below or similar to the European average, it has been ranked as the second highest rate of drug-induced mortality among the EU countries^3^. In 2017, illicit drug consumption contributed to about 2.5% of Sweden’s total disease burden, making it the tenth largest risk factor of death^4^. As ranked with the seizure quantity, the top four drugs seized in Sweden were cannabis, amphetamine, cocaine and heroin^3^. The negative impacts of drug use can result in high social costs, for instance, on conducting surveys to evaluate drug use rates, setting-up facilities for drug-related treatment services, and implementing countermeasures. Therefore, reliable data on population drug use enable public health and law enforcement agencies to efficiently allocate resources in prevention and intervention strategies on reducing drug use and its related harm.

Questionnaire-based survey is the traditional methodology for monitoring prevalence of population drug use^5^. This approach is based on self-reporting of individual usage behaviours, such as drug types, consumption frequencies, and routes of administration. However, the reliability of surveys largely depends on users’ knowledge and truthfulness of reporting, which is subjective and can be even problematic with unknown drug mixtures (e.g. mistaken alternatives to other drugs). Survey quality also demands on high numbers of respondents recruited via different survey modes (e.g. mail, online, etc.), making it costly to frequently perform and thus difficult to obtain near real-time figures. As illicit drug use is a stigmatised behaviour, objective approaches complementary to surveys allow better assessment of drug use profiles in a population. In this light, wastewater-based epidemiology (WBE) has emerged as a complementary, effective approach^6–8^. This is based on analysis of human excreted drug residues in influent wastewater, and therefore offers a direct estimation of population drug consumption within a sewer catchment. This also allows the identification and quantification of daily, short-term changes in usage patterns and the comparison of consumption across different communities over time^9^. Nowadays, the added value of WBE is recognised by the European Monitoring Centre for Drugs and Drug Addiction to yearly evaluate drug consumption among different European cities^2,5^. The Sewage Analysis Core Group Europe (SCORE) has an important role in the coordination of such annual monitoring campaigns over the last decade^5,9^.

Since its first application in 2008^6^, WBE has been continuously extended to a multitude of illicit substances and evaluations across various geographical regions from national to international scales^10–13^. In addition to assessment of spatial and temporal changes in drug use, specific events such as music festivals^14–16^ and specific settings such as prisons^17,18^ and schools^19,20^ have been also studied. In Sweden, only a few WBE studies are available^10,21–23^. The earlier-on monitoring campaigns of SCORE reported drug use by analysing six drug residues over seven-consecutive days in Umeå, Stockholm, and Gothenburg as part of a Europe-wide study^10,21^, whereas Östman et al.^22^ showed a one-day estimate of drug residues across different Swedish municipalities (without Stockholm) as a snapshot study. Recently, Löve et al.^23^ evaluated drug use among five Nordic capitals, where Stockholm wastewater samples were analysed for six drug residues, albeit seven scattered days. Each of these previous studies mainly focused on single-time estimates of population drug use. So far, studies are lacking to systematically evaluate and compare levels and patterns of drug use in different time periods between urban cities in Sweden.

In addition to the population health perspective, illicit drug residues are considered as contaminants of emerging concern in aquatic ecosystems^24,25^. Most illicit drug residues have highly polar physicochemical properties, which make them challenging to effectively remove during wastewater treatment processes^26^. While a few studies investigated the removal efficiency for a large set of micropollutants at wastewater treatment facilities in Sweden^27–29^, there remains very limited studies reporting the effluent discharge of illicit drug residues to receiving aquatic environments. Such knowledge has not been revealed in any of the previous WBE studies in Sweden. The constant usage and continuous release into the environment make illicit drug residues as pseudo-persistent contaminants^30^. In fact, a wide variety of these biologically-active substances have been detected in recipient surface waters^31–33^ and even drinking water^34^ worldwide. With psychoactive effects on humans, contamination of illicit drugs in aquatic ecosystems are then of concern, as these chemicals may also exert potent biological effects on aquatic organisms^30^. Several studies, albeit limited, have investigated ecological effects of illicit drugs, with different aquatic organisms being potentially sensitive to these compounds, as mentioned in a recent review^30^. To intervene this environmental pollution, it is therefore crucial to assess the removal efficiency of illicit drug residues at wastewater treatment plants.

With analyses of influent and effluent wastewater, our study aimed to investigate (a) the levels and patterns of daily drug consumption by the Swedish urban populations in different time periods across the year, and (b) the simultaneous removal efficiencies and weekly discharges of these drug residues to the recipient freshwater environment in Sweden. Monitoring campaigns were launched over a week, with the first one during spring while the second one during autumn; it should be noted that our monitoring windows may not necessarily reflect seasonal effects on drug use. We targeted 11 drug residues that are of public health, policy and environmental concerns^3,25^. As the major urban cities located in eastern Sweden, the sewer catchments in Stockholm and Uppsala were targeted in this study, covering about 10% of the Swedish population. WBE has never been applied before in Uppsala, the fourth-largest city in Sweden. Our study also showed, for the first time, the difference in levels and patterns of illicit drug use between Stockholm and Uppsala, and the subsequent removal efficiency and discharges of these drug residues to the aquatic environment by the Swedish urban populations. Through the inter-laboratory test of SCORE^35^, our developed analytical methods have been validated such that our results are harmonised for data comparison with other countries within this research network.

## Methods

### Chemicals and materials

All analytical standard solutions were purchased from Cerilliant (Texas, USA) at a concentration of 1.0 mg/mL (native chemicals) or 100 µg/mL (mass-labelled chemicals) in either methanol or acetonitrile. The native chemicals included amphetamine, benzoylecgonine, cocaine, ketamine, mephedrone, methamphetamine, 3,4-methylenedioxyamphetamine (MDA), 3,4-methylenedioxyethylamphetamine (MDEA), methylenedioxymethamphetamine (MDMA), 6-monoacetylmorphine (6-MAM) and norketamine. The corresponding deuterated chemicals included 6-MAM-D_6_, amphetamine-D_11_, benzoylecgonine-D_8_, cocaine-D_3_, methamphetamine-D_11_ and MDMA-D_5_. The working solution was prepared in methanol at 10 µg/mL for a native-mixture standard and at 1 µg/mL for a mass-labelled-mixture standard (internal standard, IS) stored at −20 °C in amber vials. Ultrapure Milli-Q water was generated from the Milli-Q water purification system (Milli-Q IQ 7000, Merck, Darmstadt, Germany). Concentrated hydrochloric acid (HCl), LC-grade methanol, ammonia solution (32%) and Whatman glass microfiber filters (Grade GF/D) were purchased from Sigma Aldrich. Optima LC–MS grade formic acid was acquired from Fisher Scientific (New Hampshire, USA). Oasis MCX cartridges (150 mg, 6 cc) for solid-phase extraction (SPE) and the SPE manifold with 20 ports were acquired from Waters (Massachusetts, USA).

### Sample collection

Two sampling campaigns were conducted at the major municipal wastewater treatment plants (WWTPs) in Stockholm and Uppsala over seven consecutive days, Tuesday-Monday, during spring and autumn in 2019 (Table 1). The WWTP covers about 87% of the municipality population at each location. The selected sampling weeks were considered as a ‘normal week’ without known specific events. Daily composite samples of influent and effluent wastewater were taken using flow-proportional sampling at 4 °C over the 24-h collection. Since both WWTPs have two inlet channels, a single daily influent sample was manually mixed at our laboratory for the two locations based on the wastewater flow of each channel. Besides influent wastewater, effluent wastewater was also collected over a week during the autumn campaign at both WWTPs. Collection of effluent wastewater in the first sampling campaign was unavailable. All samples were kept frozen at pH 2 adjusted by HCl (2 M) in polyethylene bottles (pre-rinsed by Milli-Q water and methanol) until further processing (~ 2 months). Both WWTPs comprise conventional treatment processes with primary and secondary clarifications. The biological treatment is performed with conventional activated sludge and after the purification. The effluent wastewater is discharged to the nearby aquatic environment, i.e., Fyrisån River in Uppsala and Lake Mälaren in Stockholm, which are freshwater resources for drinking water production.

**Table 1:** Wastewater sampling in this study.

City (WWTP)	Number of inhabitants^a^	Sampling dates^b^	Flow range (m^3^/24 h)	Sampling scheme	
				Influent^c^	Effluent^c^
Stockholm (Henriksdal)	850,000^d^	09/04–15/04/2019	240,000–270,000	X	
		15/10–21/10/2019	270,000–350,000	X	X
Uppsala (Kungsängsverket)	200,000^d^	12/03–18/03/2019	58,000–91,000	X	
		15/10–21/10/2019	43,000–58,000	X	X

^a^Data from WWTP staff incorporated with 2019 census data.

^b^From Tuesday to Monday next week. X: collected samples.

^c^Flow-proportional sampling.

^d^Cover about 87% of the municipality population.

### Sample extraction

Procedures of sample preparation with SPE were adapted from previous studies^36,37^. Briefly, filtered wastewater samples (40 mL) were fortified with IS (2 ng) and then extracted using Oasis MCX cartridges, which were pre-conditioned with methanol (6 mL), MilliQ water (5 mL) and acidified MilliQ water at pH 2 (5 mL). Sample loading was performed under vacuum with 1 drop/s. After that, cartridges were washed with acidified MilliQ water at pH 2 (3 mL) and then dried under vacuum for 40 min. Elution was performed using 2% NH_3_ in methanol (6 mL). The samples were then concentrated under pure nitrogen to 20 μL, followed by the reconstitution with MilliQ/methanol (8:2) solution for a final extract of 200 μL.

### Instrumental analysis

Final extracts were analysed for the target analytes using ultra-high-performance liquid-chromatograph (UltiMate 3000, Thermo Scientific) coupled with tandem mass-spectrometry (TSQ Quantiva, Thermo Scientific) (UHPLC-MS/MS). Data acquisition was performed using multiple reaction monitoring (MRM) and positive electrospray ionisation modes (Table S1). Ionisation source parameters and MRM transitions were optimised for each analyte, in which the two most sensitive products ions were selected for quantification and confirmation of an analyte. Chromatographic separation of the analytes was performed on a C18 column (50 mm × 2.1 mm, 1.7 μm, Acquity BEH, Waters Corporation, Manchester, UK), with mobile phases of 0.1% formic acid in MilliQ water (A) and 0.1% formic acid in methanol (B) running in a gradient of: 0–1 min, 10% B; 7 min, 35% B; 7.5–10.5 min, 100% B; 11–14 min, 10% B, at a flow rate was 0.5 mL/min. Column temperature was set at 40 °C throughout the run. Injection volume was 10 µL. Acquisition and data processing were carried out using Xcalibur and TraceFinder software (Thermo Scientific), respectively.

### Data quantification and quality control

For every batch of the instrumental analysis, chemical concentrations in the sample extracts were measured together with the 10-point calibration curve (0.1–200 ng/mL, IS 10 ng/mL) analysed for three times, with linearity 0.9973–0.9994 (Table S1). Data on positive identification of an analyte was assured based on: (a) its retention time in the samples in line with (± 0.1 min difference) that in the calibration standards and corresponding IS analyte; and (b) its MRM ion ratio in the samples equivalent to (≤ 20% relative standard deviation (RSD)) that in the calibration standards^38^. Chemical concentration was quantified with IS correction to compensate for potential chemical loss from SPE and matrix effects during instrumental analysis. IS was assigned either as its corresponding to the native chemical or with a retention time of ≤ 1 min to a native compound. Method quantification limits (MQLs) of each analyte were estimated based on its response with a signal-to-noise ratio of 10 in the quantitation transition and of 3 in the confirmation transition using the spiked and low-level wastewater samples. MQLs ranged from 1 to 100 ng/L in the wastewater matrix (Table S1).

In each SPE batch, three types of QA/QC samples were included. These were MilliQ-water as the procedure blank, Milli-Q water spiked with native analytes as the positive control of the procedure, and wastewater samples spiked with native analytes as the positive control in matrix. No target analytes were detected in the MilliQ water samples, meaning no contamination occurred during sample preparation and analysis. The procedural recovery of the target analytes using spiked Milli-Q water ranged from 94 to 110% (average) with inter-day variations (RSD%) of 4.0–19% in three different days (Table S2). Matrix spike recovery of the target analytes resulted in a range of 73–110% with inter-day precision (RSD%, *n* = 3) of 2.0–15% for influent wastewater, and in a range of 64–120% with inter-day variations (RSD%, *n* = 3) of 2.0–17% for effluent wastewater (Table S2). The resulted RSD% was within the acceptance criteria (≤ 15%) considering the guideline on bioanalytical method validation from the European Medicines Agency^39^, except for norketamine with a slightly higher RSD% in effluent wastewater (17%). This overall suggests that our method is reproducible with less than 15% variation across days for our target analytes in (waste)water matrices and 17% for norketamine in effluent matrix (Table S2). The developed analytical procedure and method have been successfully validated through the SCORE’s inter-laboratory comparison (https://score-cost.eu/monitoring/interlab/). Addition of IS prior or after filtration was tested with no substantial variations observed (average RSD%, *n* = 5, range: 6-MAM, 3.7%, 1.7–6.3%; amphetamine, 4.7%, 0.2–6.5%; methamphetamine, 5.2%, 2.1–7.0%; cocaine, 7.9%, 0.8–16%; benzoylecgonine, 6.3%, 3.7–8.1%; MDMA, 5.5%, 2.4–8.7%).

### Calculations

Estimation of drug consumption was calculated with the established equation (Eq. 1)^6^. Briefly, analyte concentrations (ng/L) measured in the samples were multiplied with daily wastewater flows (L/day) for a daily mass load (mg/day). This was further calculated with correction factors^9^ concerning the human excretion rate for a drug and the molar mass difference between a parent drug and its metabolite, resulting in a daily consumed mass load. To facilitate data comparison across communities, the estimated mass loads were divided by the number of people of the sewer catchment for population-normalised mass loads (mg/day/1000 people). These amounts were further normalised with an average reference dosage to estimate the number of drug doses consumed in the population, including 50 mg for each amphetamine and methamphetamine and 100 mg for each cocaine and MDMA^9^.

1 $${\text{Population}}\;{\text{drug}}\;{\text{consumption}}\;\left( {\text{mg}}/\text{day}/1000 \; \text{people} \right) = \frac{{{\text{Drug residue concentration}} \times {\text{flow}} \times {\text{correction}}\;{\text{factor}}}}{{{\text{Number}}\;{\text{of}}\;{\text{people}}\;{\text{of}}\;{\text{a}}\;{\text{sewer}}\;{\text{catchment}}}}$$

For each chemical, the removal efficiency (RE%) was expressed as a relative difference between the average of the daily load in influent and effluent wastewater over the seven days (Eq. 2). A positive RE% value refers to a lower chemical mass load in effluent than influent.

2 $${\text{RE}}\% = \left( {1 - \frac{{average\;daily\;load\;\left( {effluent} \right)}}{{average\;daily\;load\;\left( {influent} \right)}}} \right) \times 100\%$$

For the drug residues that are quantifiable, paired *t*-test analysis (Wilcoxon test) was performed to evaluate any significant difference (*p* < 0.05) between the two studied periods in each city and between the two cities in each studied period. Weekly consumption was illustrated using Tukey box-and-whisker plots in which the box presents 25–75% interquartile range of the dataset, the whiskers show the maximum and minimum value, and an outlier defines as 1.5 times of interquartile range. The statistical analysis and data illustration were conducted using GraphPad Prism (version 8.3.1).

## Results and discussion

### Drug residues in influent wastewater

In Uppsala, amphetamine, benzoylecgonine, cocaine, ketamine, MDMA and methamphetamine were detected each day during the spring week (Table 2). The highest mass load was measured for amphetamine (average: 110 mg/day/1000 people), followed by benzoylecgonine (62 mg/day/1000 people). Cocaine (26 mg/day/1000 people) and MDMA (17 mg/day/1000 people) were measured in relatively lower mass loads. Ketamine and methamphetamine were quantified below 5.0 mg/day/1000 people. 6-MAM, MDA, MDEA, mephedrone and norketamine were not found in any of the samples. During the autumn week, amphetamine, benzoylecgonine, cocaine, ketamine, MDMA and methamphetamine were detected every day. Mephedrone was detected from Wednesday to Friday only, with an average mass load of 9.1 mg/day/1000 people over those three days and peak on Thursday (17 mg/day/1000 people) (Figure S1). Amphetamine was again measured in the highest load with a weekly average of 150 mg/day/1000 people (excluded the outlier Monday). Benzoylecgonine, cocaine and MDMA showed lower mass loads of 75, 24 and 20 mg/day/1000 people, respectively. Mass loads of ketamine, mephedrone and methamphetamine were measured each below 5.0 mg/day/1000 people. No samples showed residues of 6-MAM, MDA, MDEA and norketamine in the autumn week. The occasional detection of mephedrone in Uppsala influent wastewater may be associated with seasonal effects on drug consumption and also, perhaps the drug market with the availability of mephedrone only during the period of our second studied campaign.

**Table 2:** Detection frequency (DF%) and daily mass loads (mg/day/1000 people) of drug residues in the influent wastewater from Uppsala and Stockholm WWTP in two different monitoring weeks.

Analytes	Campaign 1 (spring)			Campaign 2 (autumn)		
	DF%	Average (SD)	Range	DF%	Average (SD)	Range
**Uppsala**						
6-MAM	0	–	< 1.8	0	–	< 1.4
Amphetamine	100	110 (9.2)	100–130	100	150^a^ (40)^a^	100–220^a^ (2300)^b^
Benzoylecgonine	100	62 (14)	48–83	100	75 (19)	59–110
Cocaine	100	26 (7.9)	19–39	100	24 (5.6)	19–33
Ketamine	100	3.7 (2.6)	1.3–8.0	100	1.8 (0.93)	0.74–3.9
MDA	0	–	< 8.9	0	–	< 6.9
MDEA	0	–	< 0.36	0	–	< 0.28
MDMA	100	17 (6.7)	9.1–27	100	20 (11)	8.4–43
Mephedrone	0	–	< 2.9	43	9.1^c^ (5.9)^c^	2.8–17^c^
Methamphetamine	100	1.9 (0.31)	1.5–2.3	100	3.4 (0.23)	2.9–5.2
Norketamine	0	–	< 0.36	0	–	< 0.28
**Stockholm**						
6-MAM	0	–	< 1.5	0	–	< 1.8
Amphetamine	100	220 (14)	200–250	100	370^a^ (47)^a^	290–430^a^ (740)^b^
Benzoylecgonine	100	220 (71)	150–330	100	250 (80)	180–380
Cocaine	100	92 (31)	67–150	100	87 (38)	55–150
Ketamine	100	2.3 (0.49)	1.6–2.9	100	2.7 (0.65)	2.2–3.9
MDA	0	–	< 7.4	0	–	< 8.8
MDEA	0	–	< 0.30	0	–	< 0.35
MDMA	100	29 (16)	11–63	100	27 (15)	12–56
Mephedrone	0	–	< 2.4	0	–	< 2.8
Methamphetamine	100	13 (1.0)	11–14	100	16 (1.2)	14–18
Norketamine	0	–	< 0.30	0	–	< 0.35

–, not calculated due to DF 0%; SD, standard deviation.

^a^Excluded the outlier (see “Methods” for details).

^b^The outlier value on Monday.

^c^Quantifiable in samples only from Wednesday to Friday.

In Stockholm (Table 2), the detection frequency of each drug residue in both spring and autumn weeks was almost the same as that in Uppsala. The highest loads were observed for benzoylecgonine and amphetamine (each 220 mg/day/1000 people), followed by cocaine (92 mg/day/1000 people) and MDMA (29 mg/day/1000 people). Methamphetamine and ketamine were quantified in relatively very low amounts (13 and 2.3 mg/day/1000 people, respectively). 6-MAM, MDA, MDEA, mephedrone and norketamine were not found in any of the spring samples. During the autumn week, each sample of Stockholm found with residues of amphetamine, benzoylecgonine, cocaine, ketamine, MDMA and methamphetamine. This time, however, amphetamine showed the highest average mass load of 370 mg/day/1000 people (excluded the outlier Monday). Benzoylecgonine and its parent compound cocaine were found with average mass loads of 250 and 87 mg/day/1000 people, respectively. MDMA and methamphetamine were detected with an average of 27 and 16 mg/day/1000 people, respectively. Measured mass loads for ketamine were below 5.0 mg/day/1000 people. 6-MAM, MDA, MDEA, mephedrone and norketamine were not found in any of the autumn samples.

Over the two studied periods, most of the target drug residues were found at higher mass loads in Stockholm than in Uppsala (Table 2). During the spring week, amounts of benzoylecgonine (*p* < 0.02) and cocaine (*p* < 0.02) in Stockholm were three times higher compared to those in Uppsala. Similarly, MDMA (*p* < 0.05) and amphetamine (*p* < 0.02) mass loads were twice as high, while methamphetamine (*p* < 0.02) mass loads in Stockholm were more than six times higher than those in Uppsala. Only ketamine loads were slightly higher in Uppsala than in Stockholm. During the autumn week, ketamine and amphetamine mass loads were twice as high in Stockholm as in Uppsala, while amounts of benzoylecgonine (*p* < 0.02) and cocaine (*p* < 0.02) were three times higher. Five times higher mass loads were measured for methamphetamine (*p* < 0.02) in Stockholm compared to Uppsala. MDMA (*p* < 0.02) mass loads were about two time higher in Stockholm than in Uppsala during the autumn week. Our data overall suggested that drug use was dynamic in the studied populations over two monitoring campaigns. Future studies would be worthy to better investigate any seasonal effects on such (changes in) use observed in this study.

### Weekly patterns of drug consumption in two studied periods

#### Amphetamine

**Figure 1:**
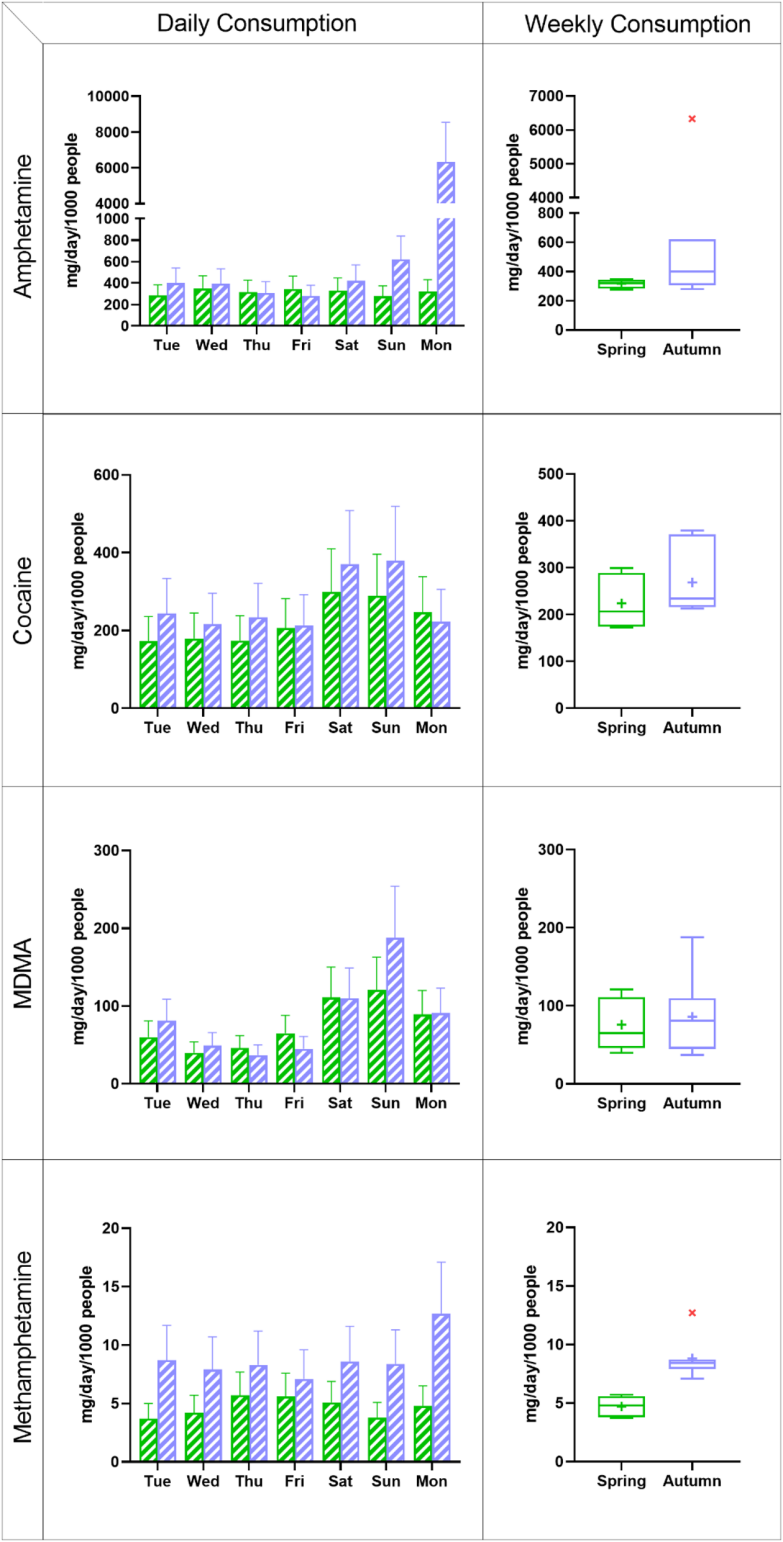
Day-to-day (bar chart) and weekly (Tukey box-and-whisker plot) drug consumption in Uppsala in two studied periods (green: campaign 1 in spring; purple: campaign 2 in autumn). Error bars taken in account the uncertainty of sampling, chemical analysis, flow measurement, excretion fraction and population as previously described^21,36,46^. In the box plot (see “Methods” for details), a red cross indicates an outlier and + represents an average value.

Amphetamine was the most consumed drug in Uppsala (Fig. 1). In spring, its consumption was consistent over the week, ranging between 280 and 350 mg/day/1000 people. During the autumn week, the consumption was mostly consistent too; however, a tenfold increase in consumption was observed from Sunday (620 mg/day/1000 people) to Monday (6300 mg/day/1000 people). Excluding the outlier on Monday, the weekly consumption of amphetamine in autumn (average: 400 mg/day/1000 people) was only slightly higher than that in spring (320 mg/day/1000 people).

**Figure 2:**
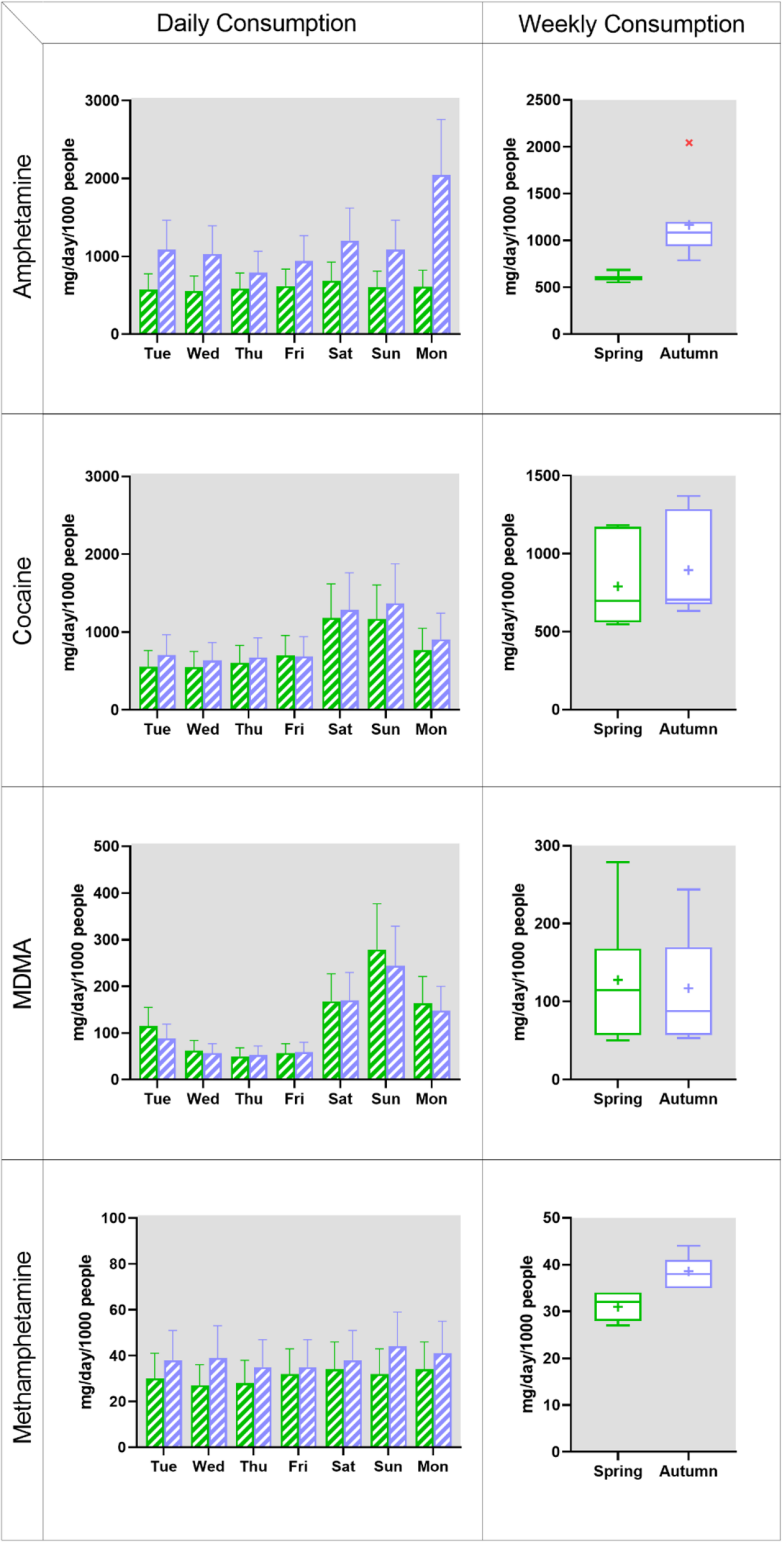
Day-to-day (bar chart) and weekly (Tukey box-and-whisker plot) drug consumption in Stockholm in two studied periods (green: campaign 1 in spring; purple: campaign 2 in autumn). Error bars taken in account the uncertainty of sampling, chemical analysis, flow measurement, excretion fraction and population as previously described^21,36,46^. In the box plot (see “Methods” for details), a red cross indicates an outlier and + represents an average value.

Over the spring week in Stockholm (Fig. 2), amphetamine consumption ranged between 550 and 690 mg/day/1000 people, showing no specific weekly usage patterns. During the autumn week, amphetamine consumption was generally between 790 and 1200 mg/day/1000 people; however, from Sunday to Monday, a twofold increase in consumption from 1100 to 2000 mg/day/1000 people was observed. Compared to spring (600 mg/day/1000 people), the average weekly consumption of amphetamine was almost twice (*p* < 0.02) as high in autumn (1000 mg/day/1000 people), even without the elevated level on Monday.

Both cities showed very similar weekly patterns with consistent day-to-day consumption over the week in the two studied periods, expect for an elevated consumption on Monday in autumn. While higher consumption of amphetamine on weekends than weekdays were reported in other European cities as reflecting recreational use^23,40^, our finding suggested that this drug appeared to be consumed rather constantly, and that regular usage was more pronounced than recreational purposes in the populations of both cities.

Among the target drug residues, amphetamine was estimated at the greatest consumption in both cities over the spring and autumn weeks. In Stockholm, available wastewater data (Table 3) generally reflected an increasing trend of weekly amphetamine consumption, from 91 mg/day/1000 people in 2011^10^ to 580 mg/day/1000 people in 2016^23^ and then 600 mg/day/1000 people during the spring week in 2019 (this study). Its consumption raised even higher later 2019, with 1000 mg/day/1000 people in the autumn (excluding the outlier on Monday). The results of both campaigns in Stockholm and Uppsala were far above the international average (86 cities) of 170 mg/day/1000 people^41^. Amphetamine was found less prevalent than cocaine in the population survey of Swedish young adults aged 17–34 years old^3^. Our wastewater results showed the opposite with more massive consumption of amphetamine than cocaine in the whole population studied. This may suggest that the prevalence of cocaine and amphetamine in young adults was different from that in overall demographics, despite the uncertain reliability of self-reporting data in surveys. The wastewater finding was consistent to the seizure data with higher quantities of amphetamine (745 kg/year) than cocaine (162 kg/year) in Sweden^3^. Particular increases in drug consumption have been observed in other WBE studies when specific events and festivals happened in catchments^42–44^. However, in our case, it remained difficult to elaborate the cause of such elevation detected on Monday in the autumn week, since there was no knowledge about official specific events held at the same time in both cities during that day and that weekend. Potential private parties cannot be excluded for understanding the observed high levels. Future studies would be of interest, for example, using chiral analysis^45^ to address if the high amphetamine level may be also attributed to a direct discharge of unconsumed substance.

**Table 3:** Comparison of drug consumption using data from WBE studies conducted in Stockholm.

Publication	Location	Year	Weekly average consumption (mg/day/1000 people)							
			Amphetamine		Cocaine		MDMA		Methamphetamine	
Thomas et al.^10^	Stockholm^a^	2011	91		190		nd		22	
Löve et al.^23^	Stockholm^a^	2016	580		550		170		62	
This study	Stockholm	2019	600^b^	1000^c^	790^b^	900^c^	130^b^	120^c^	30^b^	38^c^
	Uppsala		320^b^	400^c^	220^b^	270^c^	76^b^	86^c^	4.7^b^	8.2^c^

nd: not detected.

^a^Consumption estimated with correction factors of 2.77 (amphetamine), 3.59 (benzoylecgonine), 4.4 (MDMA), 2.44 (methamphetamine) in González-Mariño et al.^9^.

^b^Campaign 1 in spring.

^c^Campaign 2 in autumn.

#### Cocaine

Cocaine was the second most consumed drug in Uppsala (Fig. 1). During the spring week, the consumption was in a range of 170–300 mg/day/1000 people and reached its peak on the weekend (300 mg/day/1000 people each Saturday and Sunday). The consumption in the autumn week was found in a range of 210–380 mg/day/1000 people and showed a similar pattern with an increase on the weekend (Saturday: 370 mg/day/1000 people; Sunday: 380 mg/day/1000 people). The average weekly consumption in autumn (270 mg/day/1000 people) was slightly higher (*p* < 0.05) that that in spring (220 mg/day/1000 people).

In Stockholm, cocaine consumption was in a range of 550–1200 and 630–1400 mg/day/1000 people in the spring and autumn week, respectively (Fig. 2). Higher usage was observed on the weekends, with almost doubled consumption (mg/day/1000 people) from Friday (spring: 700; autumn: 690) to Saturday (spring: 1200; autumn: 1300). The average weekly consumption in the autumn (900 mg/day/1000 people) was slightly higher (*p* < 0.05) than that in the spring (790 mg/day/1000 people). The weekly consumption pattern of cocaine did not vary between the two cities and between the two seasonal weeks. Increases in cocaine consumption on weekends have been also commonly noticed and clearly showed recreational use of this drug^9^.

Previous WBE studies in Stockholm revealed a growing popularity of cocaine use over the years (Table 3). Cocaine consumption was estimated at 190 mg/day/1000 people in 2011^10^, then increasing to 550 mg/day/1000 people until 2016^23^. Our results exceeded these previous estimates with 790 mg/day/1000 people in spring and 900 mg/day/1000 people in autumn. While our recent data on cocaine consumption in Stockholm was similar to the international average (86 cities) of 790 mg/day/1000 people^41^, our Uppsala values (220; 270 mg/day/1000 people) were below that average. Both population surveys^3^ and WBE studies showed a similar increasing trend of cocaine consumption over the last few years.

#### MDMA

In both locations, MDMA showed a clear weekly pattern with a rise in consumption on the studied weekends of spring and autumn. In Uppsala, the consumption doubled between Friday and Saturday from about 65 mg/day/1000 people to more than 110 mg/day/1000 people in spring and from 45 to 110 mg/day/1000 people in autumn (Fig. 1). The average weekly consumption of MDMA in spring (76 mg/day/1000 people) and autumn (86 mg/day/1000 people) were similar in this studied population.

Similarly, in Stockholm, a strong weekly pattern was also observed, with a peak on Sunday in both the spring and autumn weeks (280 and 240 mg/day/1000 people, respectively) (Fig. 2). The average weekly consumption remained very similar over the studied periods (spring: 130 mg/day/10,000 people; autumn: 120 mg/day/1000 people). The consumption increased on weekends in both studied cities demonstrate MDMA’s use as recreational or ‘party’ drug^9^.

Together with previous WBE studies in Stockholm, no clear increasing or decreasing trends of MDMA consumption are derivable (Table 3). In 2011, MDMA was not detectable^10^. However, in 2016, its consumption has been estimated with a weekly average of 170 mg/day/1000 people^23^. Our results are slightly lower than the 2016 estimates^23^. This suggests the average consumption of MDMA remaining stable, as well as its availability in the drug market, over time (Table 3). However, it is not possible with wastewater analysis to distinguish whether or not there was the same number of users with the same drug purity, a higher number of users with decreased drug purity, or a lower number of users with increased drug purity. Compared to the international average (86 cities) of 150 mg/day/1000 people^41^, Stockholm was similar to that average and Uppsala was about half less.

#### Methamphetamine

Methamphetamine was the least consumed substance in Uppsala compared to the other three drugs (Fig. 1). Its consumption (3.7–5.7 mg/day/1000 people) was steady over the spring week, so was the autumn week (7.1–8.7 mg/day/1000 people) except for an irregular increase on Monday (13 mg/day/1000 people) (Fig. 1). The average weekly consumption in the autumn (8.2 mg/day/1000 people, excluded outlier Monday) was about two times higher (*p* < 0.02) compared to the spring average (4.7 mg/day/1000 people).

During the spring week in Stockholm, there was a consistent consumption of methamphetamine (27–34 mg/day/1000 people) with the weekly average of about 30 mg/day/1000 people (Fig. 2). This consistency was also observed in the studied week of autumn, with a higher weekly average of about 40 mg/day/1000 people, which is slightly higher (*p* < 0.02) than the amounts measured in the spring week.

Methamphetamine consumption in the autumn week exceeded those in the spring at both locations; however, the increase was less pronounced in Stockholm than in Uppsala. No specific weekly patterns in methamphetamine consumption were observed in our and a few other studied European cities^10,11^, and its usage appeared less associated with recreational purposes in contrast to MDMA and cocaine.

Our results have shown that, among the other three drugs, methamphetamine was measured at the lowest consumption level in both cities over the seasonal weeks (Figs. 1 and 2). Available wastewater data from Stockholm do not indicate a specific trend in methamphetamine use (Table 3). Between 2011 and 2016, the average weekly consumption of methamphetamine increased from 22 to 62 mg/day/1000 people^10,23^. Our results indicate a decrease by half to a weekly average of only 31 and 38 mg/day/1000 people in spring and autumn, respectively. The estimated consumption of both campaigns in Stockholm and Uppsala was far below the international average (86 cities) of 220 mg/day/1000 people^41^.

While WBE is a promising approach to estimate population drug consumption, the back-calculation process is associated with some uncertainties in different steps^21,36,37^, such as, sampling, chemical analysis, correction factors, population sizes and reference doses. The uncertainties can be managed and minimised following best practice^36^, as in our study. There could remain a particular difficulty in estimating changes in the population size, as it may vary with commuters and tourism/vacation seasons, e.g., summer. Our study selected the monitoring weeks in the months expecting reduced/low influences of tourists/vacation factors, but the possibility of weekly commuters cannot be excluded and will need future investigation based on, for instance, hydrochemical parameters^40^, pharmaceuticals^46,47^ and mobile data^48^, for improved estimates.

### Doses and market values

In both locations, the overall popularity of drug use and the drug market scale remained very similar between the spring and autumn weeks (Table 4). During the spring week in Uppsala, amphetamine (average: 6.3 doses/day/1000 people) was the most prevalent drug of choice and accounted for 67% of the total number of drug doses. Cocaine (2.2 doses/day/1000 people) was the next highly favourite drug consumed in this city, represented as 24% of the total dose. Doses of MDMA (0.76 doses/day/1000 people) and methamphetamine (0.094 doses/day/1000 people) were substantially lower and constituted a very small proportion (< 10%) of the total number of drug doses. In the autumn week, amphetamine (25 doses/day/1000 people) remained as the highest prevalent drug followed by cocaine (2.7 doses/day/1000 people), MDMA (0.86 doses/day/1000 people) and methamphetamine (0.18 doses/day/1000 people). This makes the total dose comprised 87% from amphetamine, 9% from cocaine and < 5% from both MDMA and methamphetamine. Consumption of these four drugs was estimated as 13,000 doses in the spring week and 40,000 doses in the autumn week, where showed an elevated usage of amphetamine (25,000 doses) and methamphetamine (50 doses) on Monday. The drug market in Uppsala appeared maintaining over the two studied periods, in which the greatest street value over a week was noticed for cocaine (spring: €27,000; autumn: €32,000), followed by MDMA (€15,000; €16,000) and amphetamine (€9 800; €11,000 excluding Monday).

**Table 4:** Estimated drug doses and street values over the studied periods in Uppsala and Stockholm.

	Daily doses per 1000 people (mean ± SD)	Proportion of the total daily dose (%)	Total weekly doses	Estimated weekly street value (Euro)
**Uppsala**				
Campaign 1 (spring)				
Amphetamine	5.5–7.0 (6.3 ± 0.6)	67	8900	9800
Cocaine	1.7–3.0 (2.2 ± 0.6)	24	3100	27,000
MDMA	0.4–1.2 (0.76 ± 0.3)	8.0	1100	15,000
Methamphetamine	0.073–0.11 (0.094 ± 0.02)	1.0	130	na
Sum			13,000	52,000
Campaign 2 (autumn)				
Amphetamine	5.6–130 (25 ± 45)	87	35,000	39,000
Cocaine	2.1–3.8 (2.7 ± 0.7)	9.0	3800	32,000
MDMA	0.37–1.9 (0.86 ± 0.5)	3.0	1200	16,000
Methamphetamine	0.14–0.25 (0.18 ± 0.04)	0.6	250	na
Sum			40,000^a^	87,000^b^
**Stockholm**				
Campaign 1 (spring)				
Amphetamine	11–14 (12 ± 0.8)	55	72,000	79,000
Cocaine	5.5–12 (8.0 ± 3.0)	36	47,000	400,000
MDMA	0.5–2.8 (1.3 ± 0.8)	6.0	7600	100,000
Methamphetamine	0.55–0.68 (0.62 ± 0.1)	2.8	3700	na
Sum			130,000	580,000
Campaign 2 (autumn)				
Amphetamine	16–41 (23 ± 8.0)	68	140,000	150,000
Cocaine	6.3–14 (9.0 ± 3.0)	26	53,000	450,000
MDMA	0.53–2.4 (1.2 ± 0.7)	3.0	7000	95,000
Methamphetamine	0.70–0.87 (0.77 ± 0.06)	2.2	4600	na
Sum			210,000^c^	700,000^d^

Average dose^9^: 50 mg for amphetamine, 100 mg for cocaine, 100 mg for MDMA, 50 mg for methamphetamine. Average street price in 2019^53^: amphetamine, €1.1/dose; cocaine, €8.5/dose; MDMA, €14/dose. na: not available. Compared to data obtained in the campaign 1, the autumn’s estimated doses and values increased by + 204%^a^, + 67%^b^, + 57%^c^, + 20%^d^.

Similar to Uppsala, the spring week in Stockholm showed amphetamine (12 doses/day/1000 people) as the most popular drug of choice, followed by cocaine (8.0 doses/day/1000 people) (Table 4). These two drugs contributed to about 90% of the total doses. Contrarily, MDMA (1.3 doses/day/1000 people) and methamphetamine (0.62 doses/day/1000 people) were less prevalent in the city, accounting for about 9.0% of the total number of drug doses. During the autumn week, amphetamine (23 doses/day/1000 people) and cocaine (9.0 doses/day/1000 people) were still the highly favourite drugs in this city, constituted about 94% of the total dose. Doses of MDMA (1.2 doses/day/1000 people) and methamphetamine (0.77 doses/day/1000 people) remained very low prevalent and contributed to about 5.0% of the number of drug doses. These four drugs were consumed in a total of 130,000 doses in the spring week and 210,000 doses in the autumn week with the same finding of elevated amphetamine usage (35,000 doses) on Monday as in Uppsala. With higher numbers of doses, the street value of amphetamine was also enlarged between the two studied periods (spring: €79,000; autumn: €150,000 (€114,000 excluding Monday)). Nevertheless, in Stockholm, cocaine (€400,000; €450,000) showed the strongest street value over the two seasonal weeks, whereas MDMA (€100,000; €95,000) was relatively lower in the street value.

In the two studied locations, there was a general increasing pattern of the total number of drug doses (Uppsala: + 200%; Stockholm: + 60%) and therefore the estimated street value (Uppsala: + 67%; Stockholm: + 20%) from the spring to autumn weeks, suggesting a potentially growing drug market that continuously reaches the demand on these illicit drugs in both cities over the two studied periods. While the average dose estimates allow a direct comparison of drug prevalence in the population, it should be carefully noted with a few uncertainties behind, including differences in the local market on drug purity that may vary across regions/countries and over a particular/short time period (e.g., due to shortage of precursor chemicals), in the routes of drug intake, and also in the amounts consumed by different types of users (e.g. heavy *vs.* light) and in different occasions (e.g. regular *vs.* recreational use)^6,9^.

### Effluent discharges of drug residues

Effluent samples in the autumn week were analysed for the target drug residues in order to reveal the treatment efficiency of these chemicals and their discharges into the environment. In Uppsala, each effluent wastewater was quantified for benzoylecgonine, cocaine, ketamine, MDMA, methamphetamine and norketamine (Table 5). The average daily discharge of these drug residues ranged from about 0.074 to 2.9 g/day (i.e., 0.37–15 mg/day/1000 people) in this catchment, with the lowest for cocaine and the highest for MDMA (Table 5). Mephedrone was only detectable between Thursday and Monday during the week, ranged from 0.41 to 2.5 g/day (i.e., 2.1–12 mg/day/1000 people) (Figure S1). Benzoylecgonine showed an average discharge of about 1 g/day (i.e., 4.7 mg/day/1000 people), followed by ketamine with about 0.5 g/day (i.e., 2.3 mg/day/1000 people), while discharges of methamphetamine and norketamine were even lower, in less than 0.3 g/day (i.e., 1.3 mg/day/1000 people). 6-MAM, amphetamine, MDA and MDEA were not detected in any of the effluent samples of Uppsala.

**Table 5:** Detection frequency (DF%), removal efficiency (RE%), and daily discharge in the effluent wastewater from Uppsala and Stockholm WWTPs.

Analytes	DF%	RE%	Daily discharge per 1000 capita (mg/day/1000 people)		Daily discharge in the catchment (g/day)	
			Average (SD)	Range	Average (SD)	Range
**Uppsala**						
6-MAM	0	na	–	–	–	–
Amphetamine	0	100	–	–	–	–
Benzoylecgonine	100	94	4.7 (2.1)	2.8–9.3	0.94 (0.42)	0.54–1.9
Cocaine	100	98	0.37 (0.2)	0.20–0.81	0.074 (0.039)	0.041–0.16
Ketamine	100	− 28	2.3 (0.51)	1.6–3.1	0.46 (0.10)	0.33–0.63
MDA	0	na	–	–	–	–
MDEA	0	na	–	–	–	–
MDMA	100	26	15 (5.3)	8.6–24	2.9 (1.1)	1.7–4.7
Mephedrone	71	− 31	7.2^a^ (3.7)^a^	2.1–12^a^	1.4^a^ (0.74)^a^	0.41–2.5^a^
Methamphetamine	100	64	1.3 (0.25)	1.1–1.8	0.26 (0.050)	0.22–0.36
Norketamine	100	na	1.3 (0.26)	0.99–1.8	0.26 (0.053)	0.20–0.36
**Stockholm**						
6-MAM	0	na	–	–	–	–
Amphetamine	0	100	–	–	–	–
Benzoylecgonine	100	98	4.8 (5.0)	1.7–17	4.1 (4.2)	1.4–14.1
Cocaine	100	99	0.95 (1.4)	0.13–4.2	0.81 (1.2)	0.11–3.6
Ketamine	100	− 13	3.1 (0.58)	2.4–4.3	2.6 (0.49)	2.1–3.7
MDA	0	na	–	–	–	–
MDEA	0	na	–	–	–	–
MDMA	100	21	21 (9.2)	11–36	18 (7.8)	9.4–31
Mephedrone	0	na	–	–	–	–
Methamphetamine	100	37	10 (1.7)	6.8–13	8.5 (1.5)	5.8–11
Norketamine	100	na	0.65 (0.18)	0.49–1.1	0.55 (0.16)	0.42–0.9

–: not provided due to DF 0% in effluent wastewater; na: not available as the analyte was not quantifiable in influent and/or effluent wastewaters; SD: standard deviation.

^a^Quantifiable only in samples from Thursday to Sunday.

In Stockholm, benzoylecgonine, cocaine, ketamine, MDMA, methamphetamine and norketamine were quantified in each effluent sample, in which their average daily discharge ranged from about 0.55 to 18 g/day (i.e., 0.65–21 mg/day/people) (Table 5). The highest discharge by the population was measured for MDMA, followed by methamphetamine (8.5 g/day; 10 mg/day/1000 people), benzoylecgonine (4.1 g/day; 4.8 mg/day/1000 people) and ketamine (2.6 g/day; 3.1 mg/day/1000 people). Cocaine and norketamine were measured at the lowest (below 1 g/day). No samples showed residues of 6-MAM, amphetamine, MDA, MDEA and mephedrone.

The two studied WWTPs have similar conventional treatment processes with primary and secondary clarifications, and their overall treatment performance was very similar for most of the drug residues. Both WWTPs have showed a very good removal efficiency (94–100%) for amphetamine, cocaine and benzoylecgonine, but a lower removal efficiency for methamphetamine (Uppsala: 64%; Stockholm: 37%) and MDMA (26%; 21%) (Table 5). At the WWTP of Uppsala, a negative removal efficiency was observed for ketamine (− 28%) and mephedrone (− 31%). Similar results were also noticed for ketamine (− 13%) at the WWTP of Stockholm. The observed high removal efficiencies for amphetamine, benzoylecgonine and cocaine are consistent with previous studies^31,49,50^. Although the treatment efficiency for methamphetamine have been reported in a wide range (from 25 to 100%)^49–51^, our finding still showed a good agreement with the literature values. In this study, none of the WWTPs achieved satisfactory removal efficiencies for MDMA, which is in accordance with other studies reporting limited removal of this chemical (from − 12 to 36%)^49–51^. This is also the case for ketamine with very low and even negative removal efficiencies observed^49^. This means higher amounts measured in effluent than influent wastewater, which can be explained by potential biodegradation of precursor chemicals (e.g. cleavage of conjugate metabolites) and also potential chemical re-partitioning from the particulate phase to the wastewater phase within the treatment processes^27,49^.

Our results appeared to show that for chemicals which cannot be fully removed after treatments, their occurrence pattern in the effluent wastewater simultaneously carried over from the influent wastewater. This finding was clearly observed for drugs such as MDMA, methamphetamine and mephedrone (Figure S1). Consequently, the amount of these drug residues presented such daily and weekly changes in the release of wastewater effluent, and therefore their occurrence in recipient water bodies. Effluent discharges with illicit drugs are also dependent on several aspects, such as the physicochemical properties of drug residues, types of wastewater treatment technology employed, and local environmental conditions (rainfall, temperature etc.)^49,52^. This may potentially explain the difference in the removal efficiency of methamphetamine between the two studied WWTPs. While some drug residues can survive the treatment processes at both WWTPs, some appear to be effectively removed. However, it is noteworthy that even with a high removal efficiency, the results can be due to the conversion of the drugs to (potentially hazardous) unmonitored transformation products and/or due to the adsorption of the drugs from the water phase to the solid fractions (sludge). Our effluent results overall suggested that some of these illicit drug residues were consistently discharged to the recipient freshwater body, and therefore could degrade the quality of freshwater resources, e.g., for drinking water production. Future studies would be of interest to further investigate the occurrence and environmental impact of these chemicals in the recipient water bodies, and to better understand their potential health risks to aquatic organisms.

## Conclusions

This study provided new insights into potential short-term changes in drug consumption by the Swedish urban populations. For the first time, such data are revealed in the Uppsala community, as well as the variation in drug use between Stockholm and Uppsala at different time periods. It is noteworthy that amphetamine was the most popular drug of choice in both studied cities. Compared to cocaine and MDMA, the weekly consumption pattern for amphetamine and methamphetamine was less pronounced, suggesting that their usage is less associated with recreational purposes on the weekends. The analysis of effluent wastewater showed that not all the target drug residues was efficiently removed at the WWTPs, and therefore discharges as emerging contaminants to the recipient aquatic environment, especially for MDMA, methamphetamine, ketamine and mephedrone. With wastewater sampling and analysis, our methodology demonstrated the feasibility to detect short-term variations in population drug use and in performance of wastewater treatment processes. Continuous emissions of these contaminants due to human activities (regular usages and/or recreational proposes) and their subsequent environmental impact on the recipient water quality should not be neglected in the future. Our study could be beneficial to public health and law enforcement authorities for a better understanding of drug use and availability in urban cities, as well as environmental protection agencies for potential regulation on environmental discharges of these chemicals, at local and international levels.

## Supplementary Information

Supplementary Information.
