# Supplementary material for: Mining chemical information in Swedish wastewaters for simultaneous assessment of population consumption, treatment efficiency and environmental discharge of illicit drugs
Source: Sci Rep. 2021 Jun 29;11:13510. doi: 10.1038/s41598-021-92915-4 (PMC8241857; doi:10.1038/s41598-021-92915-4)
Supplement: Supplementary file 1 — Supplementary Information. [file 41598_2021_92915_MOESM1_ESM.pdf]

Supporting Information

Mining chemical information in Swedish wastewaters for simultaneous assessment of population consumption, treatment efficiency and environmental discharge of illicit drugs

Inga Haalck[a], Paul Löffler[a], Christine Baduel[a,b], Karin Wiberg[a], Lutz Ahrens[a], Foon Yin Lai[a,*]

[a] Department of Aquatic Sciences and Assessment, Swedish University of Agricultural Sciences (SLU), Box 7050, 75007 Uppsala, Sweden.

[b] University Grenoble Alpes, IRD, CNRS, Grenoble INP, IGE, Grenoble, France.

[*]Corresponding author: email address, foonyin.lai@slu.se; postal address, Department of Aquatic Sciences and Assessment, Swedish University of Agricultural Sciences (SLU), Box 7050, 75007 Uppsala, Sweden.

Table S1: MRM transitions, retention time, linearity, MQLs and related IS used in the analytical method.

Compound	MRM (m/z) (CE, V)	MRM (m/z) (CE, V)	Retention time (min)	Linearity	MQL[a] (ng/L)	IS used
6-MAM	328/211 (27)	328/165 (39)	2.99	0.9990	5	6-MAM-D6
6-MAM-D6	334/165 (39)					
Amphetamine	136/91 (18)	136/119 (10)	2.68	0.9994	100	Amphetamine-D11
Amphetamine-D11	147/98 (20)					
Methamphetamine	150/119 (10)	150/91 (20)	3.00	0.9994	1	Methamphetamine-D11
Methamphetamine-D11	161/97 (21)					
Cocaine	304/182 (20)	304/105 (32)	5.98	0.9994	1	Cocaine-D3
Cocaine-D3	307/185 (20)					
Benzoylecgonine	290/168 (20)	290/105 (29)	4.08	0.9994	1	Benzoylecgonine-D8
Benzoylecgonine-D8	298/171 (20)					
MDMA	194/105 (23)	194/163 (10)	3.18	0.9995	1	MDMA-D5
MDMA-D5	199/165 (14)					
Mephedrone	178/119 (22)	178/160 (13)	4.17	0.9973	8	Benzoylecgonine-D8
MDA	180/105 (25)	180/163 (13)	2.96	0.9994	25	MDMA-D5
MDEA	208/163 (14)	208/135 (22)	3.95	0.9994	1	MDMA-D5
Norketamine	224/207 (13)	224/125 (26)	4.97	0.9992	1	Cocaine-D3
Ketamine	238/220 (16)	238/125 (29)	5.08	0.9994	1	Cocaine-D3

[a]method quantification limit (MQL) in influent matrix.

Table S2: Relative recovery (%) and day-to-day variation (RSD, %) of the target analytes in MilliQ water and wastewater.

	Milli-Q water spike		Influent wastewater spike		Effluent wastewater spike	
	Recovery[a] [%]	RSD[b] [%]	Matrix spike recovery[a] [%]	RSD[b] [%]	Matrix spike recovery[a] [%]	RSD[b] [%]
6-MAM	100 (95-105)	4.2	102 (98-107)	4.3	103 (99-106)	3.2
Amphetamine	94 (88-99)	5.1	107 (101-110)	5.4	99 (89-104)	8.9
Benzoylecgonine	101 (95-107)	4.5	112 (97-120)	12	106 (103-107)	1.7
Cocaine	97 (89-105)	5.9	104 (100-108)	4.3	99 (92-103)	5.7
Ketamine	109 (93-120)	10	90 (83-100)	10	82 (73-94)	13
MDA	96 (84-105)	9.3	86 (72-97)	15	80 (77-82)	3.5
MDEA	95 (85-103)	6.9	107 (95-115)	10	108 (105-111)	2.7
MDMA	98 (89-107)	6.0	105 (102-108)	3.0	104 (101-108)	3.5
Mephedrone	102 (80-120)	19	105 (93-119)	12	119 (116-120)	2.0
Methamphetamine	95 (86-103)	6.6	109 (108-111)	1.7	102 (95-107)	6.3
Norketamine	106 (91-120)	12	73 (67-77)	7.4	64 (56-77)	17

[a]average (range), n=3; [b]precision across 3 days.

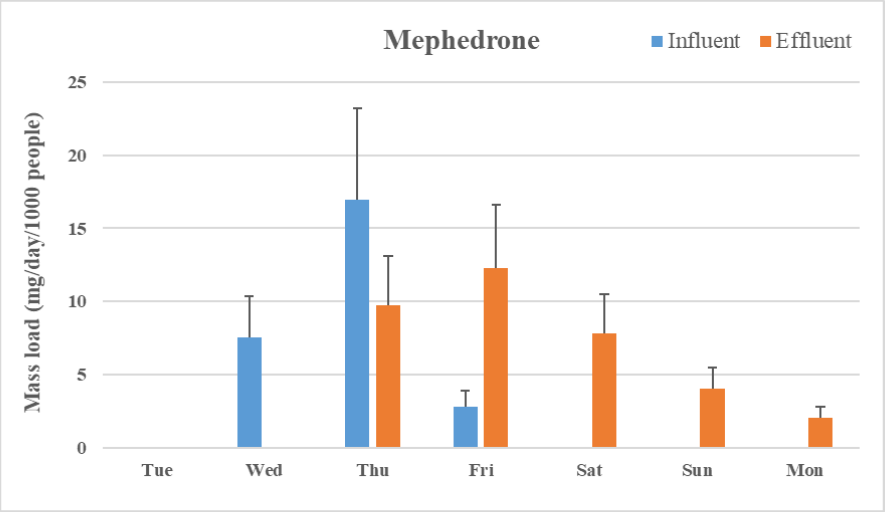

Figures S1: Daily mass loads (mg/day/1000 people) in Uppsala WWTP influent and effluent wastewaters in the second studied campaign.
